# Behavioural response of the malaria vector *Anopheles gambiae* to host plant volatiles and synthetic blends

**DOI:** 10.1186/1756-3305-5-234

**Published:** 2012-10-15

**Authors:** Vincent O Nyasembe, Peter EA Teal, Wolfgang R Mukabana, James H Tumlinson, Baldwyn Torto

**Affiliations:** 1 International Centre of Insect Physiology and Ecology, Box 30772, GPO, Nairobi, Kenya; 2 School of Biological Sciences, University of Nairobi, P. O. Box 30197, GPO, Nairobi, Kenya; 3 Center for Medical, Agricultural, and Veterinary Entomology, U.S. Department of Agriculture, Agricultural Research Service, 1700 Southwest 23 Drive, Gainesville, FL, 32604, USA; 4 Center for Chemical Ecology, Department of Entomology, Pennsylvania State University, University Park, Pennsylvania, PA, 16802, USA

**Keywords:** Sugar feeding, Host plants, *An. gambiae s.s*, Malaria vector, Attractant, Terpenoids

## Abstract

**Background:**

Sugar feeding is critical for survival of malaria vectors and, although discriminative plant feeding previously has been shown to occur in *Anopheles gambiae s.s*., little is known about the cues mediating attraction to these plants. In this study, we investigated the role of olfaction in *An. gambiae* discriminative feeding behaviour.

**Methods:**

Dual choice olfactometer assays were used to study odour discrimination by *An. gambiae* to three suspected host plants: *Parthenium hysterophorus* (Asteraceae), *Bidens pilosa* (Asteraceae) and *Ricinus communis* (Euphorbiaceae). Sugar content of the three plant species was determined by analysis of their trimethylsilyl derivatives by coupled gas chromatography–mass spectrometry (GC-MS) and confirmed with authentic standards. Volatiles from intact plants of the three species were collected on Super Q and analyzed by coupled GC-electroantennographic detection (GC-EAD) and GC-MS to identify electrophysiologically-active components whose identities were also confirmed with authentic standards. Active compounds and blends were formulated using dose–response olfactory bioassays. Responses of females were converted into preference indices and analyzed by chi-square tests. The amounts of common behaviourally-active components released by the three host plants were compared with one-way ANOVA.

**Results:**

Overall, the sugar contents were similar in the two Asteraceae plants, *P. hysterophorus* and *B. pilosa,* but richer in *R. communis*. Odours released by *P. hysterophorus* were the most attractive, with those from *B. pilosa* being the least attractive to females in the olfactometer assays. Six EAD-active components identified were consistently detected by the antennae of adult females. The amounts of common antennally-active components released varied with the host plant, with the highest amounts released by *P. hysterophorus*. In dose–response assays, single compounds and blends of these components were attractive to females but to varying levels, with one of the blends recording a significantly attractive response from females when compared to volatiles released by either the most preferred plant, *P. hysterophorus* (*χ*^*2*^ = 5.23, df = 1, *P* < 0.05) or as a synthetic blend mimicking that released by *P. hysterophorus.*

**Conclusions:**

Our results demonstrate that (a) a specific group of plant odours attract female *An. gambiae* (b) females use both qualitative and quantitative differences in volatile composition to associate and discriminate between different host plants, and (c) altering concentrations of individual EAD-active components in a blend provides a practical direction for developing effective plant-based lures for malaria vector management.

## Background

Mosquitoes need sugar for flight and other metabolic activities [1-3]. Male mosquitoes, and females of some species, depend entirely on plant nectars [1,2,4,5]. Both autogenous and anautogenous mosquitoes require carbohydrates for survival [6,7], and evidence shows that sugar ingestion plays a critical role in longevity, fecundity, flight capacity, and host-seeking behaviour [8-11]. Mosquitoes forage for sugars mainly from floral nectaries [12,13], but also from extra-floral nectaries, honeydew, plant phloem, and damaged and rotting fruits [2,14]. As such, the availability of sugar sources in the local environment is a major determinant regulating survival, the dynamics of mosquito populations and their vector potential [15,16].

Although previous studies have found scant evidence of sugar feeding in field collected *An. gambiae*, suggesting that this feeding habit rarely, if ever, occurs [17], recent studies have shown that these afrotropical malaria vectors feed intermittently on plant sugars when present in the plant habitats [10,11,17-19], and in a discriminating manner. The cues responsible for this discriminative feeding behaviour remain largely unclear. Previous studies have implicated potential fitness-related benefits (i.e. survival and fecundity) as the basis of host plant selection among malaria vectors [18]. In semi-field experiments with some *An. gambiae*-associated plants commonly found growing around homesteads in western Kenya, non-blood fed females were found to survive relatively longer and laid more eggs when presented with certain plants including *Manihot esculenta* Crantz (Euphorbiaceae), *Tecoma stans* L. (Bignoniaceae), *Ricinus communis* L. (Euphorbiaceae), and *Senna didymobotrya* Fresen (Caesalpiniaceae) [10,18,20], than when presented with other associated plants. Interestingly, these four plant species also ranked among the highly preferred host plants for the vector. On the other hand, *Lantana camara* L. (Verbenaceae), *Bidens pilosa* L. (Asteraceae), *Datura stramonium* L. (Solanaceae) and *Flaveria trinervia* Mohr (Asteraceae) performed poorly in supporting these vital life parameters and were also the least preferred host plants [10,11,18,20]. While these findings lend support to the hypothesis of benefit-based host plant selection, it was noted that *Parthenium hysterophorus* L. (Asteraceae) another highly preferred host plant, did not improve survival and fecundity [18]. Manda et al. [18] attributed this phenomenon to a possible self-medication benefit to the malaria vectors. However, the mechanism by which these malaria vectors discriminate between beneficial and non-beneficial host plants is still not clear.

Previous studies have shown that floral scents play a critical role in the location of sugar sources by mosquitoes of both sexes [2,21-24]. It would seem, therefore, that plant odours contribute to the discriminative host plant selection by females of the malaria vector *An. gambiae*. From a management perspective, if these chemicals could be identified, and particularly those from plants which are highly attractive to mosquitoes, they can be used as lures in mosquito surveillance and control programs. Despite this potential, little is known about the composition of the volatiles released from these host plants attractive to mosquitoes [23]. Their capacity to attract mosquitoes of both sexes and of varying physiological states and ages [2,3,25] makes plant-based attractants more appealing as a surveillance and control tool. In this study, we define the chemical basis by which *An. gambiae* females discriminate between different host plants. We used electrophysiological, behavioural and chemical analysis to demonstrate that olfactory cues mediate the discrimination of three differentially preferred host plant species for sugar feeding by females of this species. Our study also demonstrated that altering blend ratios of electrophysiologically-active components can increase their attractiveness to female mosquitoes, to the point of being more attractive than intact plants, thereby providing a practical direction for developing plant-based lures for this disease vector.

## Methods

### Mosquitoes

Mosquitoes used in this study were obtained from a colony reared at the International Centre of Insect Physiology and Ecology (*icipe*), Duduville campus, Nairobi, established in 2001 from blood-fed and gravid *An. gambiae s.s*. caught at Mbita Point, western Kenya. They were reared at a mean temperature and relative humidity of day, 31°C, 52% RH and night, 24°C, 72% RH; and a reversed circadian rhythm of light (03:01-15:00) and darkness (15:01-03:00). The adults were maintained on a diet of human blood three times per week, along with glucose (6% solution *ad libitum*) (Sigma®) continuously available on filter paper. Fully engorged females were allowed to lay eggs on funnel-shaped filter paper placed over oviposition cups (4 cm diameter, 2 cm depth) inside the cages. Eggs were collected and dispensed into plastic trays (25 cm long × 20 cm wide × 14 cm high) filled to a depth of 8 cm with distilled water. Upon hatching, larvae were reared in these trays at densities of 100-150/tray and fed fish food (Tetramin®) three times daily (the total amount of food provided was 0.3 g tetramin/100 larvae/day). Pupae were collected from rearing trays and transferred to standard 30 × 30 × 30 cm mesh-covered cages with access to water and 6% glucose solution *ad libitum*. Newly emerged adult females intended for use in bioassays and electrophysiological experiments were kept on 6% glucose solution only (no blood meal) until they were 2-3 days old. The mosquitoes were placed in 15 × 15 × 15 cm mesh-covered cages and starved of glucose solution for 6 h prior to the experiments, with only water available, in wet cotton wool.

### Plant material

The three plant species used in this study were selected on the basis of their relative preference for sugar feeding from previous studies [10,11]. They included *Ricinus communis* (Voucher number 2011/107; Euphorbiaceae; highly preferred by mosquitoes and with high sugar content), and two other plants differentially preferred by the vector; *Parthenium hysterophorus* (Voucher number 2011/108; Asteraceae; highly preferred but with low sugar content) and *Bidens pilosa* (Voucher number 2011/105; Asteraceae; less preferred and with low sugar content) [10,11]. The plant seedlings were obtained from *icipe* station at Mbita Pt., Homa Bay County, Kenya, and they were transplanted into potting soil and then maintained in a screenhouse at the Duduville campus under ambient conditions (day, 24°C, 52% RH; night, 25°C, 52% RH). The plants were watered daily and used at flowering stage (20-30 extrafloral buds with exudates oozing from some of the extraflorals for *R. communis*; 30-40 clusters of flowers in the case of *P. hysterophorus* and 15-20 flowers for *B. pilosa*). They were transferred to the laboratory at least 3 h prior to bioassays and allowed to acclimatize under red fluorescent light (preliminary results showed the plants recovered stable night volatile release rates within 3 h of transfer into a dark bioassay room).

### Dual choice olfactometer assays

**Figure 1:**
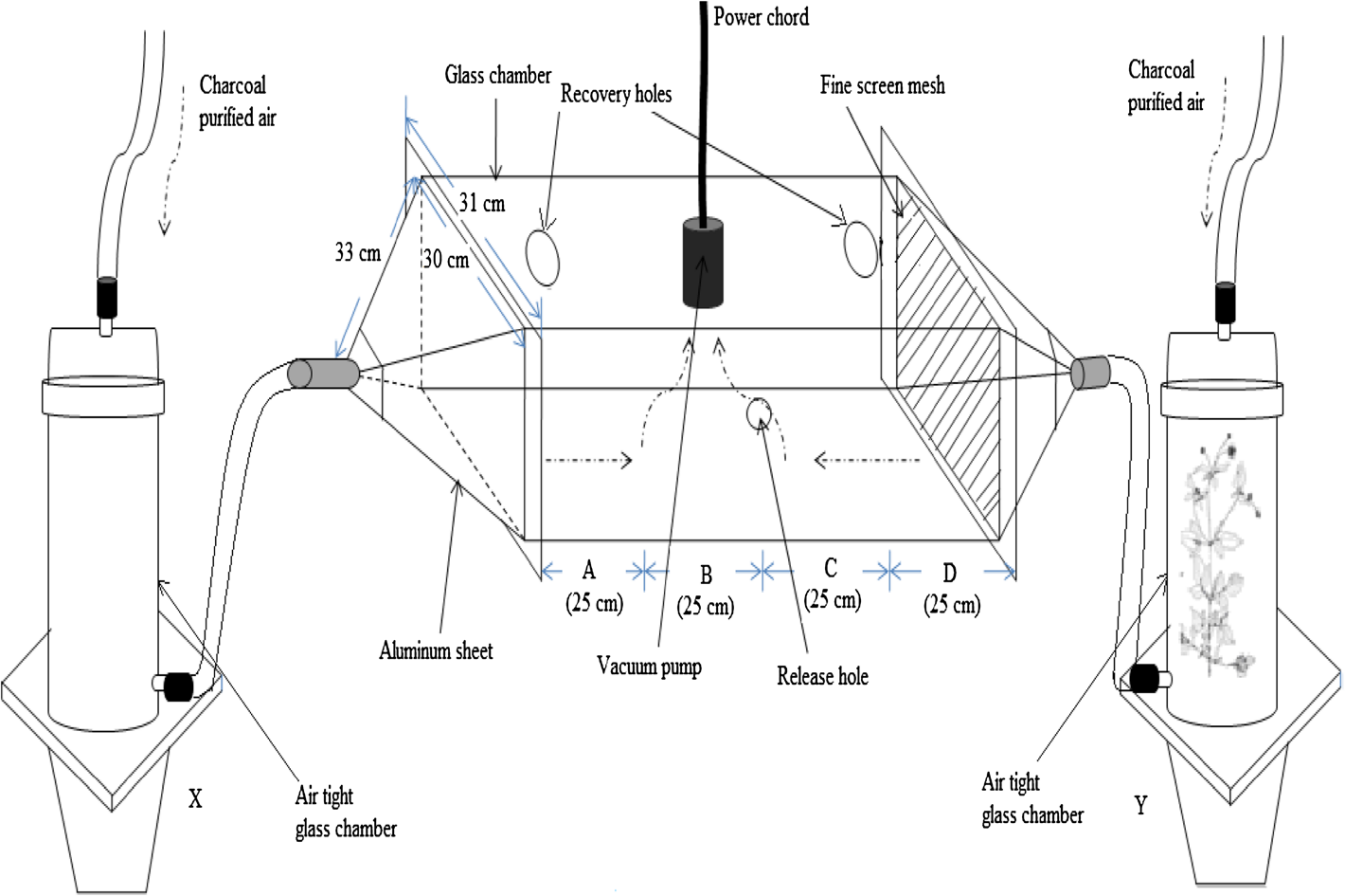
**A schematic drawing of the dual choice olfactometer (not drawn to scale).** X and Y are the glass chambers that hold intact plant while the broken arrows points to the direction of air flow. Air currents were drawn bidirectionally through the central chamber by applying a vacuum in the center of the chamber as shown in the figure. The tapering ends are made of aluminum sheet while the main olfactometer chamber is made of glass perspex.

Bioassays were carried out using a dual choice olfactometer shown in Figure 1, similar to that described by Torto et al. [26]. Briefly, air from a compressed air tank was first purified by passing it through activated charcoal and then humidified by passing through distilled water. The air flow was then split into two halves. One half was passed through a glass chamber (ARS, Gainesville, FL, USA®) enclosing a potted plant (test) and into one arm of a 30 × 30 × 100 cm olfactometer at a flow rate of 350 ml/min, while the other half was passed through an empty glass chamber (control) into the other arm of the olfactometer at the same flow rate. A vacuum line powered by a fan pulled air from the centre of the olfactometer at 700 ml/min. Two 40-W red fluorescent bulbs placed above the centre of the olfactometer illuminated the test arena evenly. Female *An. gambiae* were assayed for host-plant attraction to the three plant species in separate assays as follows: (a) each plant species was assayed against a control (air), and (b) the three plant species were then assayed against each other in pair wise comparisons. The positions of the test plants and the control in the olfactometer arms were randomized between runs. Ten female mosquitoes were released at the centre of the olfactometer in each bioassay, and this was replicated five times per plant species with different potted plants used in each bioassay. A steady flow of charcoal-filtered purified humid air was passed over the test (with plant) and control chambers and into the olfactometer (mean temperature 24°C, and 72% RH maintained in the bioassay room). The study was conducted between 14:00-20:00 (this time was arrived at following preliminary experiments which showed optimal activity), and each bioassay lasted for 10 min. Mosquitoes landing in zone A and D (within 25 cm from either ends of the olfactometer, Figure 1) were deemed to have responded to either the control or test odours while those staying between zones B and C (25 cm from the release point on either sides) were considered non-respondents. The number of mosquitoes responding to the test and control odour sources was counted in each run.

#### Sugar analysis

One gram of leaves and flowers of *R. communis, P. hysterophorus and B. pilosa* including extraflorals in *R. communis* were separately macerated slowly in 2 ml pyridine (Sigma®) for 3 days. These were then derivatized with 100 μl pyridine and 100 μl *N*-Methyl-bis trifluoro acetamide (MBTFA) (Sigma®) at 60°C for 1 h. The products were analyzed by splitless injection using an Agilent technologies-7890 gas chromatograph coupled to a 5975C inert XL EI/CI mass spectrometer (EI, 70 eV, Agilent, Palo Alto, California, USA) (GC-MS) equipped with an HP-5 column (30 m × 0.25 mm ID × 0.25 μm film thickness, Agilent, Palo Alto, California, USA), with helium as the carrier gas at a flow rate of 1.2 ml/min. The oven temperature was held at 35°C for 5 min, then programmed to increase at 10°C/min to 280°C and maintained at this temperature for 10 min. Plant sugars were identified by comparison of spectra of their trimethylsilyl derivatives with library data (Adams2.L, Chemecol.L and NIST05a.L) and with those of authentic standards (see sources and purity under chemical section below). The amount of sugar present in the different plant parts was quantified based on peak area comparison with those of authentic standards.

### Collection of volatiles

Volatiles released from the intact aerial parts of *P. hysterophorus, R. communis* and *B. pilosa* were collected by enclosing an intact plant in an air-tight glass chamber and passing air through it (at a flow rate of 350 ml/min) into adsorbent Super-Q traps (30 mg, Analytical Research System, Gainesville, Florida, USA). Talento timer based volatile collection system (Analytical Research System, Gainesville, Florida, USA) was employed in capturing volatiles released at night (19:00-06:59). The Super-Q traps were eluted with 200 μl GC/GC-MS-grade dichloromethane (DCM) (Burdick and Jackson, Muskegon, Michigan, USA) and the eluate was stored at -80°C until used.

### Analysis of volatiles

Coupled GC-EAD analysis of volatiles was carried out using a Hewlett-Packard (HP) 5890 Series II gas chromatograph equipped with an HP-1 column (30 m × 0.25 mm ID × 0.25 μm film thickness, Agilent, Palo Alto, California, USA) with nitrogen as the carrier gas at 1 ml/min. Volatiles were analysed in the splitless mode at an injector temperature of 280°C and a split valve delay of 5 min. The oven temperature was held at 35°C for 3 min, then programmed at 10°C/min to 280°C and maintained at this temperature for 10 min. The column effluent was split 1:1 after addition of make-up nitrogen gas for simultaneous detection by flame ionisation detector (FID) and EAD. For EAD detection, silver-coated wires in drawn-out glass capillaries (1.5 mm I.D.) filled with Ringer saline solution [27] served as reference and recording electrodes. Antennal preparations were made by first cutting the base of the head and distal end of antenna with a scalpel. The reference electrode was connected to the base of the head, and the recording electrode was connected to the cut tip of the antenna. The analog signal was detected through a probe (INR-II, Syntech, Hilversum, the Netherlands), captured and processed with a data acquisition controller (IDAC-4, Syntech, the Netherlands), and later analyzed with software (EAG 2000, Syntech) on a personal computer. An aliquot (5 μl) of the Super Q-trapped volatile extract of each plant was analyzed using fresh female antennae in at least three replicate runs.

For identification, the volatile extracts were analyzed using coupled GC-MS and oven conditions described above. GC-EAD-active components were identified both by comparing their mass spectral data with those recorded in the Mass Spectral Library NIST/EPA/NIH 2005a and by co-injection with authentic standards. For quantification, the peak area of each component was compared to that of an internal standard (corresponding to 29.35 ng methyl salicylate).

#### Chemicals

The synthetic standards of the following EAG-active compounds were used: hexanal (Aldrich, 98%), β-pinene (Chemika, 99.5%), β-ocimene (Chemika, (*Z*)-β-ocimene = 27%, (*E*)-β-ocimene = 67% and allo-ocimene = 6%), limonene (Sigma), (*E*)-linalool oxide (Aldrich), and (*E*)-β-farnesene (Bedoukian Research, CT, USA). The following sugars were used: (L-rhamnose, Sigma, 99%; D-(+)-galactose, Sigma, 99%; D-(-)-fructose, Sigma, 99%; sucrose, Sigma, 99.5%; maltose, Sigma, 99%; and D-(+)-glucose, Sigma, 99.5%).

### Bioassay with chemicals

The dual-choice olfactometer described above was used to test behavioural responses of female *An. gambiae* to synthetic standards of each of the six EAD-active components and a blend constituted from them. Five doses of each of these compounds were prepared at a concentration of 0.05, 0.1, 0.2, 0.4 and 0.8 ng/μl in pentane (see Additional file 1 for release rates). These were dispensed by applying 200 μl of each of the prepared doses onto 100 mg of Luna dental roll (Roeko®, Langenau, Germany), which were then left for 30 min at room temperature to allow the solvent to evaporate. The controls consisted of 100 mg dental rolls impregnated with 200 μl of the solvent (pentane) only. Each dose was tested against the control and replicated five times with freshly impregnated dental rolls used each time. The most attractive doses were then tested against an intact *P. hysterophorus* (the most attractive plant). A blend comprised of optimal doses of the individual components (i.e. 0.2 ng/μl hexanal, 0.2 ng/μl β-pinene, 0.2 ng/μl D-limonene, 0.1 ng/μl (*E*)-β-ocimene, 0.2 ng/μl (*E*)-linalool oxide and 0.1 ng/μl (*E*)-β-farnesene, referred to as Blend B henceforth) was prepared and evaluated against the solvent in the olfactometer. Dose response studies were performed by halving and doubling the amounts of the individual compounds. Blend A contained half the optimal doses (0.1 ng/μl hexanal, 0.1 ng/μl β-pinene, 0.1 ng/μl D-limonene, 0.05 ng/μl β-ocimene, 0.1 ng/μl (*E*)-linalool oxide and 0.05 ng/μl (*E*)-β-farnesene), while the amounts in Blend C contained twice the amounts contained in Blend B (see Additional file 2 for release rates). A blend comprising the natural amounts of EAD-active components in *P. hysterophorus* (i.e. 0.02 ng/μl hexanal, 0.4 ng/μl β-pinene, 0.2 ng/μl D-limonene, 0.9 ng/μl β-ocimene, 0.08 ng/μl (*E*)-linalool oxide and0.3 ng/μl (*E*)-β-farnesene, referred to as Blend X) was also prepared and tested against the solvent and against Blend C (the most attractive blend) in the olfactometer. Ten female mosquitoes were released at the centre of the olfactometer as described above. The experiment was replicated five times per dose with fresh females and sample used in each bioassay. The number of mosquitoes responding to the test and control odour source was counted for each dose. The three blends were tested against potted *P. hysterophorus* in a dual-choice olfactometer.

### Statistical analysis

A preference index (*PI*) for all the dual choice assay data was calculated according to the formula:

(1) $$PI=\left[\left(SS-NSS\right)/\left(SS+NSS\right)\right]x100$$

where *SS* is the number of mosquitoes responding to test odours and *NSS* the number of mosquitoes responding to control odours [28]. The *PI* would be zero if equal numbers of mosquitoes were found in each side of the chamber and ± 100 if all mosquitoes preferred one side of the chamber. A positive value indicates a majority of the mosquitoes responding to test odours, while a negative value indicates the converse.

Within each group, count data was subjected to a chi-squared test to test if the response differed from zero. Potential differences in sugar content and volatile release rates between the three plant species were detected by log-transforming the quantities and subjecting the transformed data to one-way ANOVA and Tukey post-hoc tests. All statistical analysis was carried out using R software [29].

## Results

### Olfactometer assays

**Figure 2:**
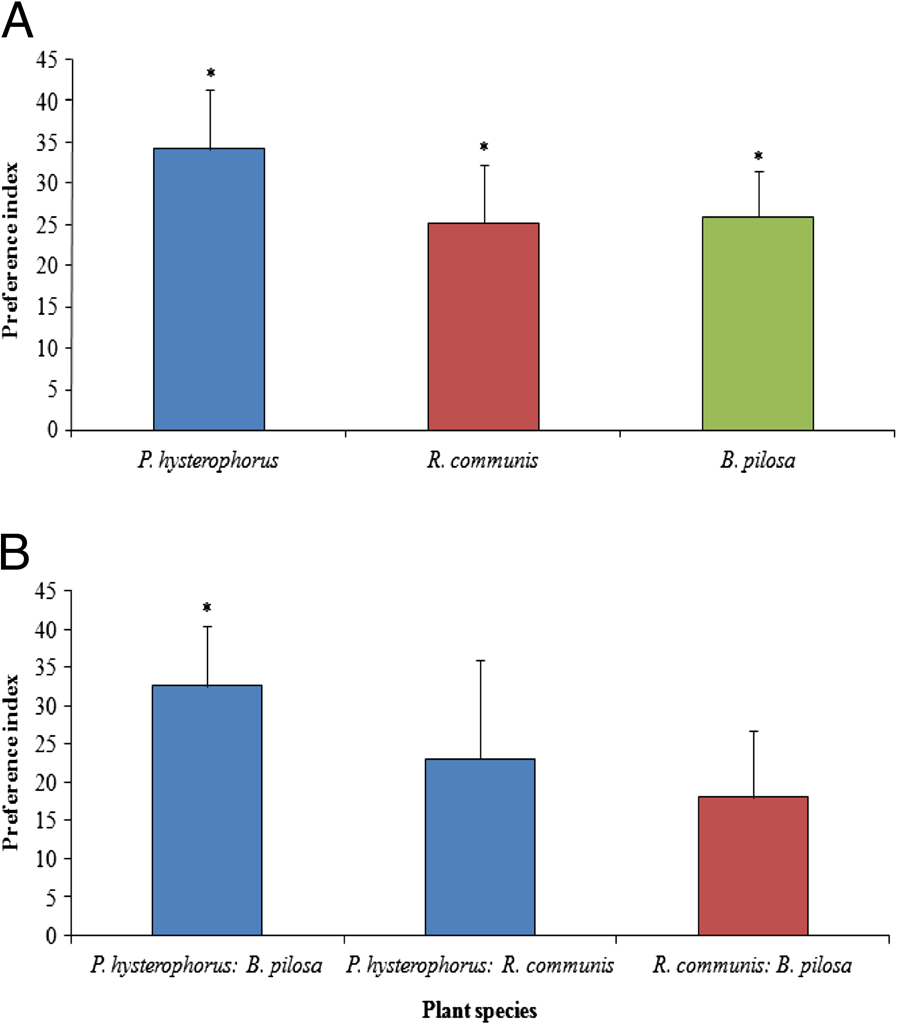
**Olfactometer responses of *An. gambiae *to odour of intact plants. A**) intact plant odours versus blank control; **B**) plant odours from different species expressed as *Preference Index* (*PI*) ± SEM. Positive response indicates preference for the first odour source. The asterisks indicate the significance levels with * = significant at 0.05, and ** = significant at 0.01.

All three host plant species were significantly more attractive to female mosquitoes (*P. hysterophorus*: +34.2%, *χ*^*2*^ = 5.44, df = 1, *P* < 0.05; *R. communis*: +25.2%, *χ*^*2*^ = 4.33, df = 1, *P* < 0.05; and *B. pilosa*: +25.9%, *χ*^*2*^ = 4, df = 1, *P* < 0.05) (Figure 2A) than the control. In paired assays *P. hysterophorus* was more attractive than *B. pilosa* (+32.6%, *χ*^*2*^ = 3.93, df = 1, *P* < 0.05) but not significantly more attractive than *R. communis* (+23%) (Figure 2B). There was no difference in attractiveness between *R. communis* and *B. pilosa* (+18%).

### Sugar analysis

Six plant sugars comprising glucose, galactose, fructose, rhamnose, sucrose and maltose were detected in the flower and leaf extracts of the three plant species. There was a significant difference in the mean sugar content of *R. communis, P. hysterophorus* and *B. pilosa* (F_(2, 105)_ = 2.62, *P* < 0.01 respectively). *Ricinus communis* extraflorals had the highest amount of all the sugars while *B. pilosa* leaves had the least amount (Table 1). Maltose was the most abundant sugar among all the three plants while rhamnose was the least abundant. Between the three plant species, maltose was significantly higher in *R. communis* than *P. hysterophorus* and *B. pilosa* (F_(5, 102)_ = 48.18, *P* < 0.001).

**Table 1:** **Mean sugar contents of leaves and extraflorals/flowers of *R. communis, P. hysterophorus *and *B. pilosa***

**Plant species**	**Sugar**	**Amount in leaves ± SEM (ng/mg)**	**Amount in flowers ± SEM (ng/mg)**
*R. communis*	Glucose	129.51 ± 33.65	365.93 ± 65.67
	Galactose	701.24 ± 119.08	17.46 ± 5.73
	Rhamnose	9.46 ± 2.08	44.57 ± 11.29
	Fructose	198.63 ± 50.36	196.65 ± 53.76
	Sucrose	225.03 ± 51.81	170.93 ± 33.01
	Maltose	4826.39 ± 345.55 (ab)	6785.31 ± 462.99 (a)
	**Total**	**6084.26** ± **164.47 (a)**	**7580.85** ± **842.40 (a)**
*P. hysterophorus*	Glucose	392.11 ± 55.61	392.11 ± 46.61
	Galactose	491.73 ± 33.81	463.83 ± 48.95
	Rhamnose	43.69 ± 4.97	42.59 ± 10.94
	Fructose	202.79 ± 50.77	84.08 ± 15.42
	Sucrose	85.68 ± 15.65	79.75 ± 24.87
	Maltose	3500.91 ± 242.33(ab)	1382.71 ± 168.21 (b)
	**Total**	**4716.91 ± 265.33(ab)**	**2445.07 ± 101.49 (b)**
*B. pilosa*	Glucose	112.29 ± 40.28	334.52 ± 53.31
	Galactose	381.85 ± 68.12	102.24 ± 34.87
	Rhamnose	49.09 ± 11.63	47.43 ± 15.66
	Fructose	113.13 ± 37.28	192.50 ± 51.88
	Sucrose	89.93 ± 22.71	87.32 ± 5.20
	Maltose	1037.05 ± 291.53 (b)	1623.07 ± 202.81 (b)
	**Total**	**1783.34 ± 228.35 (b)**	**2387.08 ± 119.80 (b)**

The values denoted with letter a, b and ab are significantly different between the three plants.

### Analysis of volatiles

**Figure 3:**
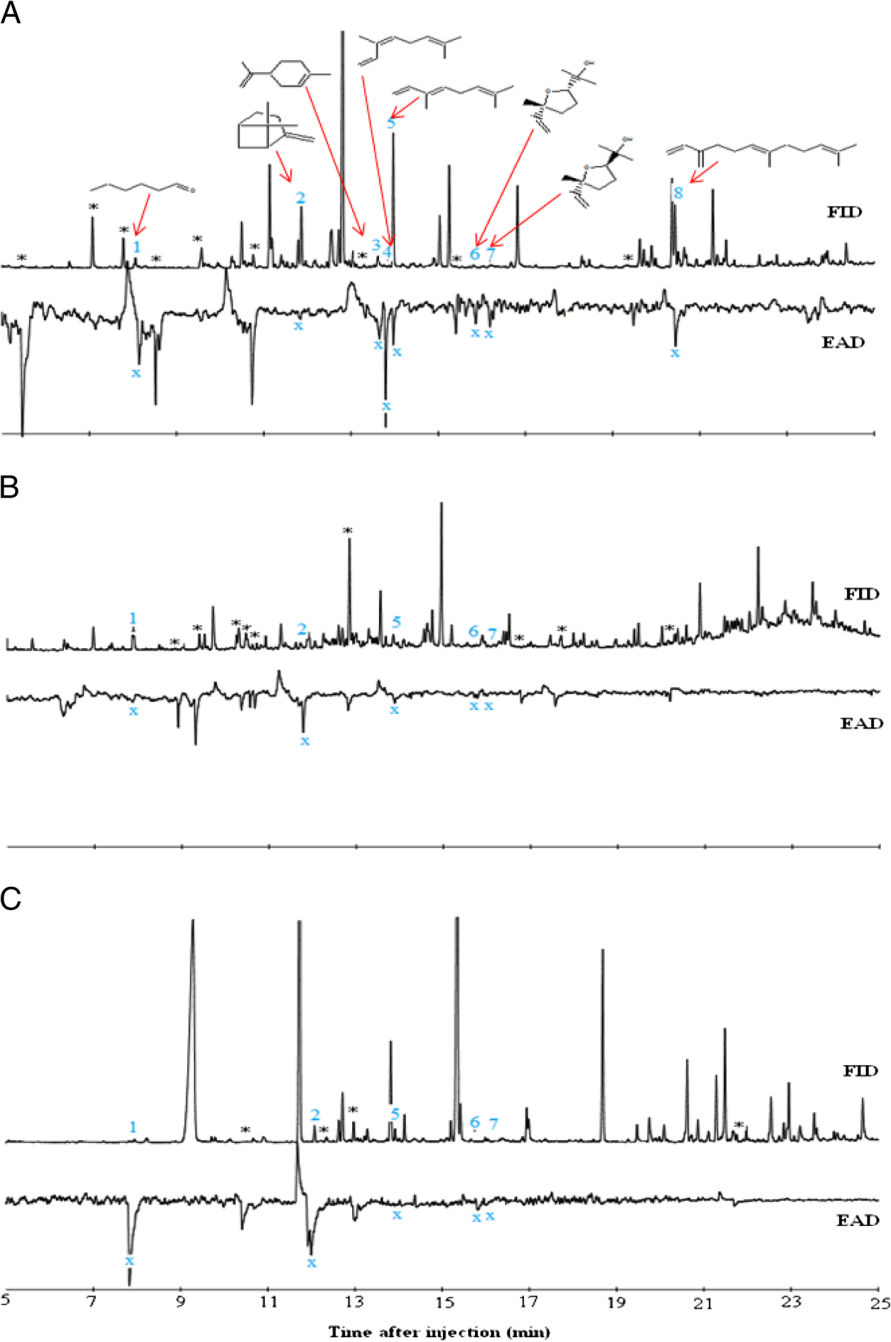
**Coupled GC-electroantennographic responses of *An. gambiae *to volatiles of the three host plant species. A**) *P. hysterophorus*; **B**) *R. communis*; and **C**) *B. pilosa*. The EAD-active compounds include hexanal (1), β-pinene (2), D-limonene (3), (*Z*)- β-ocimene (4), (*E*)- β-ocimene (5), (*Z)*-linalool oxide (6), (*E*)-linalool oxide (7) and (*E*)- β-farnesene (8) with their corresponding antennal response labelled as x.

Between six and fifteen EAD-active components were detected in the volatiles of each of the three host plants by the antennae of female *An. gambiae*. Six of these were consistently detected by the mosquito antennae in repeated runs, and these were identified as hexanal, β-pinene, limonene, (*E*)-β-ocimene, (*E*)-linalool oxide and (*E*)-β-farnesene (Figure 3: A, B, and C). Of these, limonene and (*E*)-β-farnesene were specific to *P. hysterophorus*. Both (*Z*)- and (*E*)*-* forms of β-ocimene and linalool oxide were present in all three plant species and these components also had EAG activity, but *R. communis* lacked detectable amounts of (*Z*)-β-ocimene.

**Figure 4:**
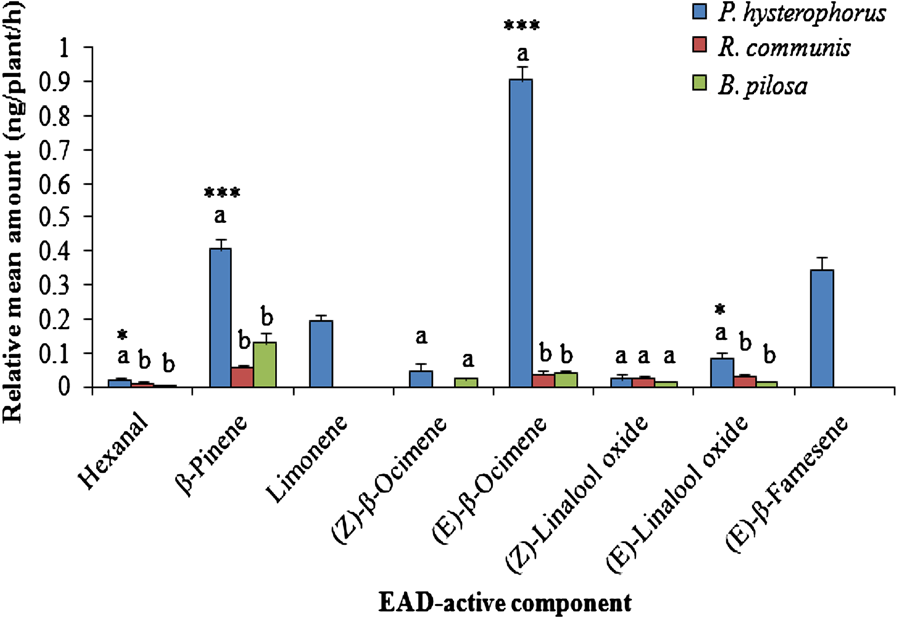
**Relative amounts of EAD-active components in volatiles of the three species. ***P. hysterophorus; R. communis * and *B. pilosa* expressed as mean ± SEM. Bars capped with different letters are significantly different between the three plant species. The asterisks indicate the significance levels with * = significant at 0.05, and *** = significant at 0.001.

The ANOVA showed that there was an overall difference between the three plants in the amount of each EAD-active volatiles produced (F_(4, 40)_ = 12.42, *P* < 0.001). The Tukey tests (as indicated in Figure 4), showed that *P. hysterophorus* produced more volatiles than the other two other plants.

### Bioassays with chemicals

**Figure 5:**
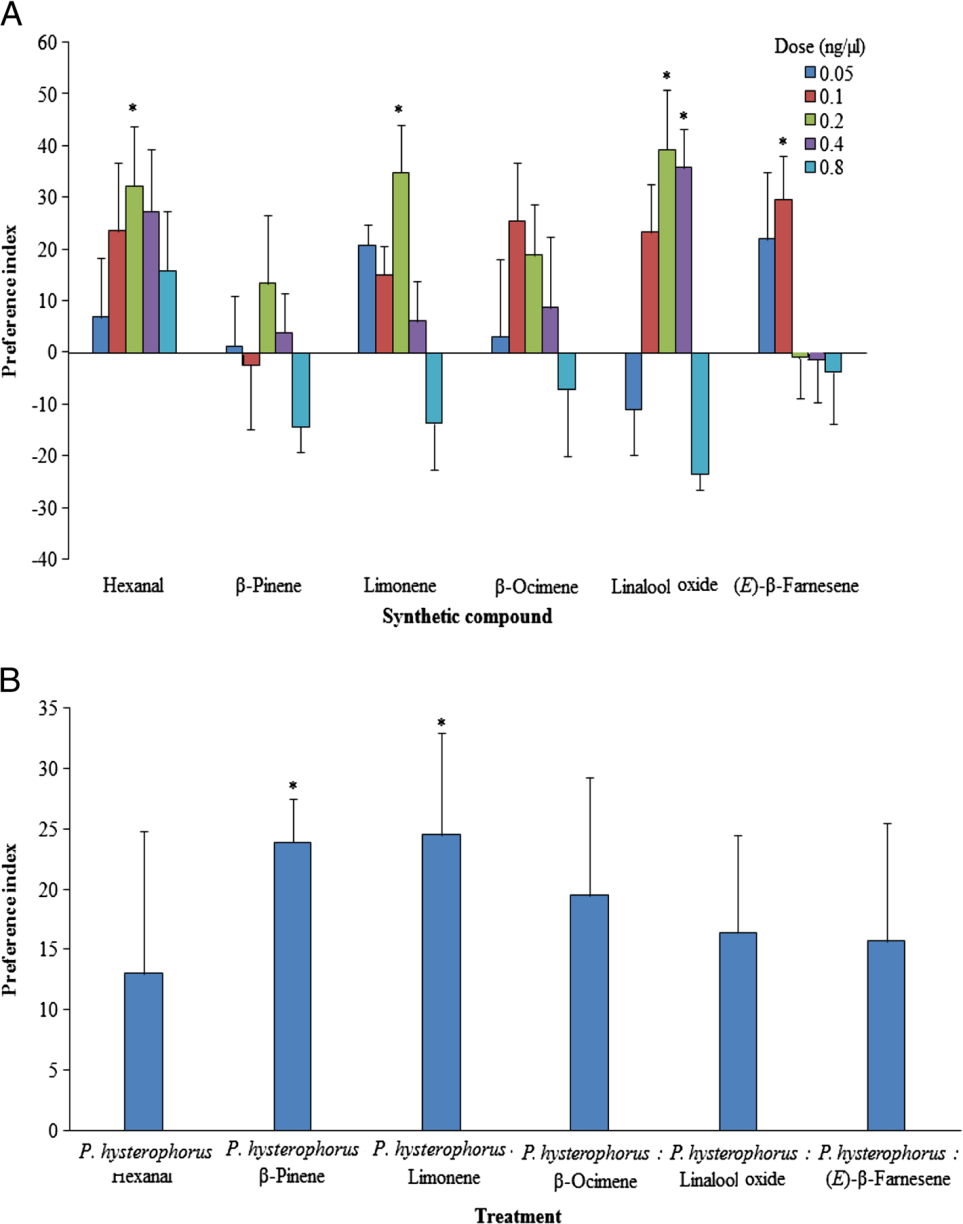
**Olfactometric response of *An. gambiae *to synthetic compounds of EAD-active components. A**) Individual EAD-active volatile components at different concentrations against solvent and **B**) intact *P. hysterophorus* volatiles against optimal attractive doses of EAD-active volatile components expressed as PI ± SEM. Positive response indicate preference for the first odour source. The asterisks indicate the significance levels with * = significant at 0.05, and ** = significant at 0.01.

**Figure 6:**
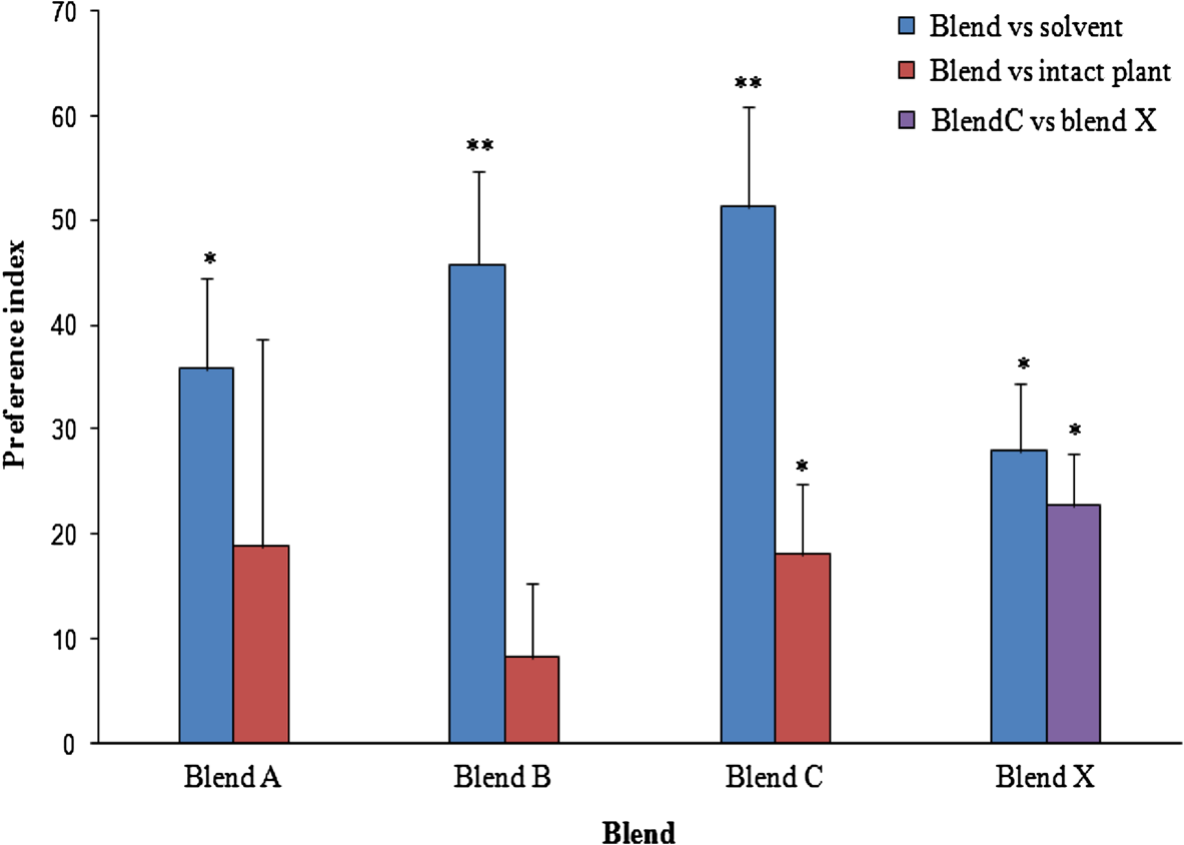
**Olfactometric responses of *An. gambiae *to synthetic blend of EAD-active volatile components against pentane and intact *P. hysterophorus *expressed as mean *PI *± SEM.** Positive *PI* indicates preference for the first odour source. The asterisks indicate the significance levels with * = significant at 0.05, and ** = significant at 0.01.

Olfactometer assays showed that females responded to all six compounds tested singly or in a blend in a dose-dependent manner. Of the six EAD-active components, hexanal, β-pinene, D-limonene and (*E*)-linalool oxide, were highly attractive at 0.2 ng/μl, while β-ocimene and (*E*)-β-farnesene were optimally attractive at 0.1 ng/μl (Figure 5A; Table 2). While hexanal remained attractive at all five doses, females demonstrated avoidance behaviour to higher doses of the other five compounds. We also noted that (*E*)-linalool oxide was significantly attractive at 0.4 ng/μl (Table 2). However, the intact *P. hysterophorus* was significantly more attractive than β-pinene and limonene but not so when compared to the other four compounds (Figure 5B). Dose response studies showed that all three concentrations of the optimal blend (blends A-C) were attractive to females, compared to the control, but to varying levels (Blend A: +35.9%, *χ*^*2*^ = 5.23, df = 1, *P* < 0.05; Blend B: +45.8%, *χ*^*2*^ = 9.09, df = 1, *P* < 0.01; and Blend C: +51.3%, *χ*^*2*^ = 10.76, df = 1, *P* < 0.01) (Figure 6). The most attractive blend (Blend C) was 20% more attractive than the intact plant (*χ*^*2*^ = 5.23, df = 1, *P* < 0.05). On the other hand, Blend X representing the natural blend of the six components in the volatiles of *P. hysterophorus* was 27.9% more attractive than the solvent (*χ*^*2*^ = 5.82, df = 1, *P* < 0.05) but 22.8% less attractive compared to Blend C (*χ*^*2*^ = 4.67, df = 1, *P* < 0.05).

**Table 2:** ***PI *and *t*-values of optimally attractive concentration of the individual EAD-active compounds**

**Compound (dose, ng/μl)**	**PI (%)**	**DF**	***χ***^***2***^	***P*-value**
1. Hexanal (0.2)	32.2	1	4.45	< 0.05
2. β-Pinene (0.2)	13.3	1	0.86	0.355
3. Limonene (0.2)	34.9	1	5.57	< 0.05
4. (*E*)-β-Ocimene (0.1)	25.4	1	3.13	0.077
5. (*E*)-Linalool oxide (0.2)	39.3	1	6.08	< 0.05
6. (*E*)-Linalool oxide (0.4)	35.8	1	5.57	< 0.05
7. (*E*)-β-Farnesene (0.1)	29.7	1	3.93	< 0.05
8. *P. hysterophorus*: β- Pinene	23.9	1	6.09	< 0.05
9. *P. hysterophorus*: Limonene	24.5	1	3.93	< 0.05

## Discussion

Results from the dual-choice olfactometer assays showed that the malaria vector *An. gambiae* responded to odours from all the three suspected host-plant species. The data further showed the existence of odour-based host-plant discrimination by this malaria vector. Our results corroborate those previously reported for other mosquito species responding to plant odours in olfactometer assays [30-35] and which was later demonstrated in *An. gambiae* by Foster and Takken [25]. Discriminative plant-feeding behaviour of this malaria vector was previously considered only in the light of potential benefits to the mosquitoes [10,18,25], and little attention has been paid to the contribution of olfactory cues to observed host-plant selection. Here we present evidence of odour-based host plant discrimination in *An. gambiae*. These findings lend support to previous reports, which indicated that plant odour in addition to visual cues and the accessibility of nectar, influences the acceptance of a plant as a sugar source by mosquitoes [36-39]. Indeed, the fact that a synthetic blend C was more attractive than the most preferred host plant indicates that odour perception is a key to selection of suitable feeding sources by this mosquito.

Our results also show that of the three plant species, although *R. communis* has a superior sugar content, *P. hysterophorus* still possesses a substantive sugar reserve contrary to the findings by Manda et al. [18]. While these findings lend support to the hypothesis of potential benefit as the primary basis for host plant selection, they also point to an evolutionary mosquito-plant interaction in which the mosquitoes are able to identify potential host plants using their odour plumes.

Although it has been postulated that terpenoids and aromatics are responsible for mosquito-host plant interactions [23], limited attempts have been made to identify the specifically active plant volatile components attractive to mosquitoes. In our study we documented using electrophysiological and behavioural assays, that *An. gambiae* detects and responds to hexanal, β-pinene, limonene, β-ocimene, (*E*)-linalool oxide and (*E*)-β-farnesene. Of these, only hexanal is not a terpene. Interestingly, (*E*)-linalool oxide has previously been reported as an attractant for *Culex pipiens*[40] and has been shown to generate a prolonged tonic response in a number of odour receptors of *An. gambiae*[41] while ocimene has been shown to be detected by the antennae of *Aedes aegypti*[42]. Although aldehydes have not been previously implicated as cues utilized by *An. gambiae,* our study indicates that hexanal is utilized by the malaria vector in host plant location. This observation is contrary to that demonstrated for culicines [43], which are not attracted to hexanal. Overall, our results demonstrate the significance of both aldehydes and terpenes in host plant selection.

Our results show that mosquitoes only detect a select number of compounds released by the plants and that often they may involve components present in low quantities. For example, the isomers of linalool oxide, which were detected by the antennae of the mosquito and were present in relatively low quantities. These results are consistent with previous findings, which indicate that the chemoreceptors in the antennae of any insect species can detect only specific components of the released volatiles and most often the most dominant volatile components are not necessarily the most important in terms of behaviour [44-46].

Dose–response studies further illustrate the significance of odour concentration in mosquito responsiveness. At lower doses, individual terpenes elicited an attractive response to females, while at higher doses, avoidance behaviour was observed. The dose-dependent attractive response was also observed when blends of the compounds were tested. The volatile composition of the three plants differed significantly both qualitatively and quantitatively. These observations emphasize the significance of concentration of essential volatile compounds beside their quality in host plant location by *An. gambiae.* Thus, it seems probable that odour sources releasing low to moderate amounts of the volatiles signal an attractive host, whereas sources with relatively high release rates would signal a marginal or non-preferred host. It is also possible that at high concentration, these plant volatiles have an arresting effect, signaling to the mosquitoes that they have arrived at the host. This observation is consistent with the view that the mechanisms of host-plant selection in insects are largely a matter of gradation and balance between chemicals rather than clearly defined and different cues [47].

The finding that combination of the individual compounds results in increased attraction and that blend C in our laboratory assays is even more attractive than the intact *P. hysterophorus* and the natural blend (blend X) is intriguing. This points to possible association of this blend with the odour of a more attractive host plant by *An. gambiae* and also stresses on the significance of odour ratios in host plant selection by the mosquito. The role of specific and general plant odours in host plant selection has been widely investigated in agricultural pests [48], but little is known about their role in nectar feeding insects of medical importance. This study presents an opportunity to further evaluate the role played by these plant compounds in the ecology of malaria vectors and possibly come up with new intervention measures against these and other disease vectoring mosquitoes.

Current research into odour-based technology as a surveillance and control strategy has emphasized attraction to human odours. The limitation of these odour baits is that they target only a specific subgroup of mosquitoes that are ‘blood thirsty’. On the contrary, plant based odours offers an opportunity to target both male and female mosquitoes of different physiological states and ages [9]. There is therefore the need to develop phytochemical baited traps that can be deployed for outdoor sampling of the malaria vector *An. gambiae*. Our study attempts to close this gap towards developing these plant-based attractive odours as a new approach to the management of malaria vectors and other mosquito vectors of diseases [49,50]. However, competition from background flora odours and the more preferred human host could reduce the effectiveness of such plant-based odour baits. These challenges can be overcome either by placing the traps away from competing natural phytochemicals and raising their release rates well above background levels as suggested by Foster [3] or by incorporating human synergistic compounds in the formulated blend which would minimize trapping of non-target insects and increase the competitive advantage of the plant-based odour baited traps.

## Conclusions

These results demonstrate the role of odours in discriminative malaria vector-host plant attraction, and they show that females use both qualitative and quantitative differences in volatile composition to associate with host plants. The increased preference for the formulated blend shows the potential for exploitation of phytochemical attractants in surveillance and control of malaria vectors.

## Competing interests

The authors declare that they have no competing interests.

## Authors’ contributions

Conceived and designed the experiments: VON PEAT JHT BT. Performed the experiments: VON. Analyzed the data: VON WRM BT. Wrote the paper: VON PEAT JHT WRM BT. All authors approved the final version for submission.

## Supplementary Material

Additional file 1 Olfactometer release rates of synthetic standards Olfactometer release rates for optimal doses of individual EAD-active synthetic standards. Click here for file

Additional file 2 Olfactometer release rates of blend components Olfactometer release rates of EAD-active synthetic components of blend A, B and C. Click here for file
