# Supplementary figures and images for: Behavioural response of the malaria vector Anopheles gambiae to host plant volatiles and synthetic blends
Source: Parasit Vectors. 2012 Oct 15;5:234. doi: 10.1186/1756-3305-5-234 (PMC3523964; doi:10.1186/1756-3305-5-234)

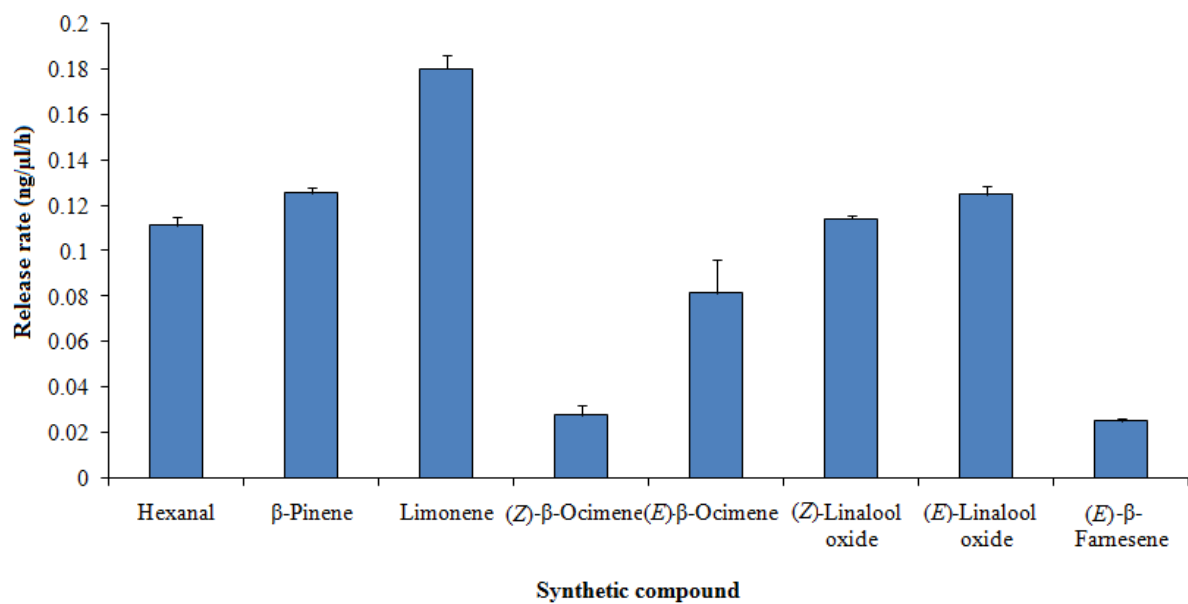

Supplement: Additional file 1 — Olfactometer release rates of synthetic standards Olfactometer release rates for optimal doses of individual EAD-active synthetic standards. [file 1756-3305-5-234-S1.pdf]

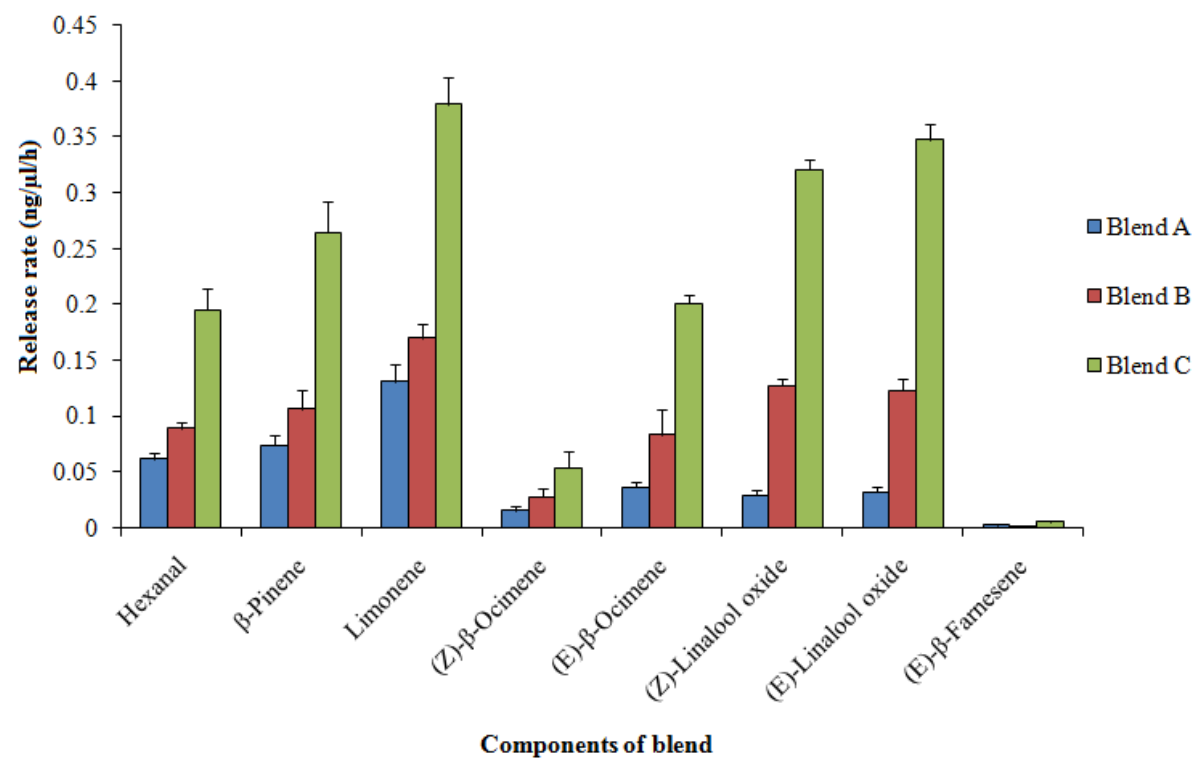

Supplement: Additional file 2 — Olfactometer release rates of blend components Olfactometer release rates of EAD-active synthetic components of blend A, B and C. [file 1756-3305-5-234-S2.pdf]
